# A method and system for generating a combination of psychological testing scales

**DOI:** 10.1098/rsos.241859

**Published:** 2025-07-09

**Authors:** Yuanye Cao, Xiangyu Shan, Huailing Ma, Xu Liu, Junwu Zhu, Yuanyuan Gao

**Affiliations:** ^1^ Medical College, Yangzhou University, Yangzhou, Jiangsu, People’s Republic of China; ^2^ School of Information Engineering, Yangzhou University, Yangzhou, Jiangsu, People’s Republic of China

**Keywords:** tree-structured probability judgement, psychological testing scale, dynamic combination generation method

## Abstract

Given the inherent limitations of traditional psychological testing scales in terms of breadth and specificity, both comprehensive and individual assessment scales offer distinct advantages. However, challenges persist because of their complementary deficiencies in practical applications. This article argues that intricate content optimization of scales is not necessary; instead, it conceptualizes comprehensive scales and individual assessment items as a multi-label classification problem. By employing a hierarchical framework, it becomes possible to achieve overall optimization of psychological testing scales. To this end, the paper introduces an algorithm designed to generate combinations of psychological scales, optimizing both comprehensive and individual assessments. This optimization is realized through a series of methodological steps, including the analysis of historical positive diagnosis data, the calculation of item probability indices, the dynamic adaptation of test content, the sequencing of items and the construction of a hierarchical scale system. Simulation experiments demonstrate that this approach enhances the efficiency and accuracy of psychological testing, particularly in diagnosing moderate to severe symptoms. However, the algorithm exhibits relatively lower accuracy for mild symptoms owing to their lower positive rate. The proposed algorithm significantly improves the optimization of psychological testing scales, particularly excelling in the assessment of moderate symptoms.

## 1. Introduction

In recent years, societal attention to adolescent mental health issues has grown significantly. While mental health issues can be quantified to some extent through standardized instruments such as self-report measures, e.g. the Minnesota Multiphasic Personality Inventory (MMPI) and the Personality Assessment Inventory (PAI), subjective expressions remain a critical component of comprehensive evaluations [1]. Therefore, it is crucial to adopt scientific methodologies that integrate both standardized tools and subjective assessments for measuring and assessing adolescent mental health. Psychological assessment tools, including adaptive and traditional scales, serve as effective instruments for quantifying mental states in both research and clinical practice. However, despite their widespread use, traditional psychological testing scales still face several critical challenges that require innovative solutions, particularly in *optimization of scale content, real-time dynamic adjustment, and multi-symptom assessment*.

Existing research on psychological testing scale optimization primarily focuses on two aspects. First, scale simplification: traditional psychological testing scales are typically categorized into two types—comprehensive psychological scales and single-item assessment scales [2]. Comprehensive scales contain multiple items suitable for multidimensional mental health evaluation but may lack specificity and efficiency. Conversely, single-item scales focus on assessing individual symptoms with high specificity and precision, yet fail to address the need for rapid diagnosis of multiple symptoms. Traditional scales, responses on a Likert scale [3], such as ‘somewhat agree’ and ‘strongly agree’, are generally assigned numerical values ranging from 1 to 5. These values are then aggregated into composite indexes (e.g. scale scores), which are used to summarize the overall response pattern and are subsequently analysed using statistical techniques. However, it is important to note that statistical techniques are usually applied to the resultant scale scores (e.g. sum scores of items) rather than individual items. This distinction is crucial for understanding how psychological data are processed and interpreted. In addition, individual response styles (e.g. extreme responding, midpoint responding) can influence the interpretation of these scores.

Second, cross-cultural adaptability: through localized scale adaptation to meet psychological assessment needs across different cultural contexts. For instance, the Riskslim algorithm [4] has been applied to simplify the Generalized Anxiety Disorder Scale (GAD-7) and validated across multinational samples. However, despite progress in static scale optimization and item simplification, significant gaps remain in dynamically adjusting scale content during testing to accommodate different symptoms and cultural variations. Therefore, there is a pressing need to enhance cross-cultural adaptability through real-time dynamic adjustments and optimized scale content.

To address these challenges, we introduce an optimized algorithm for generating combined scales. This algorithm builds on existing approaches such as computer adaptive testing and first derives probability indices and rankings from historical positive diagnostic data. It then integrates comprehensive scales with single-item assessment scales that target the same symptoms through a tree structure, while also calculating the positive rate in real time. This approach aims to enhance the efficiency and accuracy of psychological assessments by dynamically adapting to individual response patterns and prioritizing relevant items.

The main contributions of this article are summarized as follows:

(i) *scale content optimization and sequencing:* to resolve the limitations of traditional scales in item ordering and prioritization, we propose a novel probability index-based ranking method that enhances scale flexibility and adaptability through optimized question sequencing;
(ii) *multi-symptom assessment with real-time adjustment*: to overcome existing scales’ shortcomings in achieving both broad coverage and high specificity, we develop a hybrid psychological testing scale. This integrated approach combines multiple testing scales in a tree structure, enabling dynamic calculation of positive rates based on subjects’ real-time selections while addressing multi-symptom assessment needs and cross-cultural mental health evaluation requirements; and
(iii) *empirical validation*: the algorithm’s effectiveness is rigorously verified through experimental procedures, with detailed analysis of its performance under various screening conditions demonstrating validity in multi-symptom contexts.

The remainder of this article is structured as follows: §2 reviews pertinent literature on algorithms for psychological testing scales; §3 presents a formal representation of the psychological scale system model; §4 elaborates on the proposed algorithm in detail; §5 assesses the algorithm’s effectiveness through experimental validation. Finally, §6 concludes the article with a summary.

## 2. Related work

The increasing awareness of mental health issues has led to a growing interest among health management researchers in using psychological scales for collecting psychological data and conducting preliminary assessments of mental health concerns. Traditional psychological scales typically consist of a large number of items, enabling a comprehensive evaluation of a patient’s condition. However, they are often cumbersome and time-consuming to administer. As a result, researchers have been exploring methods to reduce the number of items in these scales while maintaining measurement accuracy, thereby improving their practicality and usability. For example Leite *et al*. [5], proposed a scale simplification technique based on the ant colony optimization (ACO) algorithm, which produced a 22-item abbreviated version of the Diabetes-39 scale for assessing patients’ quality of life. Simulation studies demonstrated that the ACO algorithm outperformed conventional item selection methods, such as maximum factor loading and maximum test information, highlighting its effectiveness in scale simplification.

Similarly Mwamikazi *et al*. [6], developed the T-PREDICT dynamic electronic questionnaire, which aimed to decrease the number of items in the Myers–Briggs type indicator questionnaire. Experimental results revealed that T-PREDICT successfully reduced the number of items without increasing the error rate. The methodologies used in scale design within mental health research have gradually evolved from traditional statistical approaches to more advanced classification algorithms. For instance Zhang *et al*. [7], explored techniques to improve predictive accuracy by applying various classification models—such as multi-way decision trees, logistic regression, support vector machines and random forests—to Symptom Checklist (SCL) - 90 scale data. The results demonstrated that the logistic regression classifier achieved the highest performance in predicting students’ mental health.

Furthermore Boina *et al*. [8], proposed a data mining approach that integrates multi-way trees with artificial neural networks (ANN), capitalizing on the interpretability of multi-way trees alongside the classification accuracy of ANN. Recent advancements in machine learning [9–12] technologies have significantly enhanced psychological assessment, demonstrating superior accuracy and efficiency compared to traditional methodologies. Colledani *et al*. [12] highlighted the benefits of machine learning decision trees (ML-DTs) in mental health evaluations, with research findings indicating that the diagnostic accuracy of ML-DTs (ranging from 0.71 to 0.75) exceeded that of traditional Diagnostic and Statistical Manual of Mental Disorders algorithms (0.69) and receiver operating characteristic curves (0.70 to 0.71), effectively classifying patients with depression even with limited information, thus underscoring the potential of ML-DTs to integrate conventional psychological measurement techniques and provide efficient assessment tools.

In this section, we have reviewed several key studies in the field of mental health assessment. However, it is important to note that existing research primarily focuses on the design and optimization of static psychological testing scales, with limited attention given to the dynamic adaptation of scale content based on real-time feedback from test subjects. While some studies have incorporated machine learning techniques to improve the accuracy of mental health predictions, challenges persist in enhancing testing efficiency and ensuring diagnostic reliability.

To overcome these challenges, we propose a method to improve the effectiveness of psychological scales by computing a probability index for each question and ranking them based on their significance. Additionally, it suggests the integration of comprehensive psychological testing scales with individualized assessment scales into a tree structure that computes the positive rate based on the real-time responses of the evaluated subjects. This approach aims to amalgamate comprehensive scales with individual assessment tools to provide a holistic evaluation of a patient’s mental state, thereby improving testing efficiency while maintaining measurement accuracy and reducing the number of items required.

## 3. Methodology

### 3.1. Model establishment

Given the challenges associated with current mental health surveys, it is clear that evolving social environments and psychological issues have rendered traditional fixed-order psychological testing scales insufficient for timely updates that accurately reflect changes in individual psychological states. This limitation hinders the ability to conduct rapid and precise assessments. To address this issue, this study presents an optimization model that dynamically enhances psychological scales by computing probability indices for individual items and ranking them accordingly.

Let the historical dataset of psychological scales be represented as D={C,S,R}, where the comprehensive scale $C=\left\{c_{1},c_{2},\ldots ,c_{n_{c}}\right\}$ and the single assessment item scale $S=\left\{s_{1},s_{2},\ldots ,s_{n_{s}}\right\}$ each comprise $n_{c}$ and $n_{s}$ items, respectively. The set of symptom labels $R=\left\{r_{1},r_{2},\ldots ,r_{m}\right\}$ contains m symptom labels. The user’s selection set for assessment items is defined as $U=\left\{u_{1},u_{2},\ldots ,u_{n_{u}}\right\}$, where $u_{j}\in \left\{0,1,2,3,4\right\}$ indicates the user’s choice corresponding to the options ‘none, mild, moderate, severe, and extreme’ score. An item is considered positive when the user selects ‘moderate, severe, or extreme’.

An item can be denoted as $t_{n}=\{r_{i},u_{n},text\}$, where $r_{i}$ represents the symptom label targeted by the item, $u_{n}$ is the option selected by the user, and text signifies the content of the question. The items within the comprehensive scale can be expressed as $c_{n}=\{r_{i},u_{n},text\}$, where the items $r_{i}$ correspond to the same condition. Similarly, the items in the single assessment scale can be represented as $s_{n}=\{r_{i},u_{n},text\}$, where the items $r_{i}$ within the same single assessment scale are identical.

Let the set of items associated with the condition label $r_{i}$ in the comprehensive scale be denoted as $T_{i}^{C}$. Given that the number of items corresponding to each condition label in the comprehensive scale is relatively limited, sorting is unnecessary. Let $k_{i}$ represent the number of items corresponding to each condition label $r_{i}$ in the comprehensive scale, such that $k_{i}=\left|T_{i}^{C}\right|$ and $k_{i}\leq n_{c}$. For example, if a comprehensive scale contains 10 items measuring anxiety on a 5-point Likert scale, then $k_{i}=10$ for the anxiety condition label. This parameter helps determine the number of items available for each symptom, ensuring that the algorithm can prioritize the most relevant questions based on historical data. Also, if a user selects three out of five items indicating ‘moderate’ or higher symptoms (e.g. depression) with a preset threshold of 50%, the algorithm will generate additional questions from the single-item assessment scale to further evaluate the severity of depression. This step-by-step approach ensures assessments remain both efficient and tailored to user responses. The items in the single assessment scale are specifically designed to evaluate a particular condition. Let the set of items used to assess condition $r_{i}$ in the single assessment scale be $T_{i}^{S}$, thus $T_{i}^{S}\subseteq S$.

The first-order probability index for the condition label $r_{i}$ in the comprehensive scale is denoted as $P_{\left(i\right)}$, with $N_{C}$ representing the total number of individuals using the comprehensive scale C. The number of positive diagnoses for the condition label $r_{i}$ is $N_{\left(i\right)}$. The first-order probability index $P_{\left(i\right)}$ is calculated as the ratio of the number of positive diagnoses for that condition label to the total number of individuals using the comprehensive scale, expressed mathematically as follows:


(3.1) $$\begin{array}{lcr} & P_{\left(i\right)}=\frac{N_{\left(i\right)}}{N_{C}}. & \end{array}$$

The set of first-order probability indices is denoted as $P=\left\{P_{\left(1\right)},P_{\left(2\right)},\ldots ,P_{\left(m\right)}\right\}$, where each $P_{\left(i\right)}$ corresponds to a condition label $r_{i}$.

However, it is important to note that both probability index implicitly assumes the independence of psychological symptoms, meaning that the probability of a symptom being positive is not influenced by the presence of other symptoms. While this assumption simplifies the calculation, it may not fully capture the complexity of psychological states, as extensive empirical research has shown that psychological symptoms often exhibit intricate comorbidity patterns. For example, anxiety and depression are frequently highly correlated, and psychotic symptoms may co-occur with paranoid tendencies in nonlinear ways. To address this limitation, future iterations of the algorithm could incorporate conditional probabilities or multivariate statistical methods to model the dependencies between symptoms. For instance, Bayesian networks or machine learning approaches could be employed to capture the complex relationships among psychological symptoms, thereby improving the explanatory power of the proposed probability metrics.

The second-order probability index for the item $s_{j}$ in the single assessment scale is represented as $P_{i,j}$, with $N_{S}$ indicating the total number of individuals using the single assessment scale S. The number of individuals diagnosed as positive after completing this item is $N_{i,j}$. The second-order probability index $P_{i,j}$ for the item $s_{j}$ in the single assessment scale is defined as the ratio of the number of positive diagnoses for that item to the total number of individuals using the single assessment scale, specifically expressed as:


(3.2) $$\begin{array}{lcr} & P_{i,j}=\frac{N_{i,j}}{N_{S}}. & \end{array}$$

The set of second-order probability indices is denoted as $P_{i}=\left\{P_{i,1},P_{i,2},\ldots ,P_{i,n_{s}}\right\}$, where each $P_{i,j}\in P_{i}$ corresponds to the item $s_{j}$ in the single assessment scale, with the condition label being $r_{i}$.

To address the limitations of traditional psychological testing scales, which often feature a large number of items in comprehensive scales but exhibit broad coverage, and a limited number of items in single assessment scales with narrow coverage, leading to inefficiencies in confirming positive symptoms and resulting in time-consuming and labour-intensive processes, a tree structure index model for scales has been developed. This model systematically generates test questions by aligning the optimized comprehensive scale with the symptom labels in the single assessment item scale and constructing a tree structure.

The root node of the tree is denoted as $D^{\prime }$, with its child nodes representing the symptom labels $r_{i}$ corresponding to the questions $T_{i}^{C}$ in the comprehensive scale, while the leaf nodes comprise the set of single assessment item questions $S_{i}^{\prime }$ associated with the symptom label $r_{i}$. The tree can be represented as an ordered pair (V,E), where V denotes the set of nodes and E signifies the set of edges.

The node set V includes the root node $D^{\prime }$, the optimized comprehensive scale question set $T_{i}^{C}$, and the optimized single assessment item question nodes $S_{i}^{\prime }$:


(3.3) $$\begin{array}{lcr} & V=\{D^{\prime }\}\cup T_{i}^{C}\cup S_{i}^{\prime }. & \end{array}$$

The edge set E illustrates the connection relationships between nodes, indicating that the root node $D^{\prime }$ is connected to each optimized comprehensive scale question set $T_{i}^{C}$:


(3.4) $$\begin{array}{lcr} & E_{D^{\prime }}=\{(D^{\prime },T_{i}^{C})|T_{i}^{C}N_{S}\subseteq C^{\prime }\}. & \end{array}$$

Each optimized comprehensive scale question set $T_{i}^{C}$ is linked to its corresponding single assessment item question set $S_{i}^{\prime }$:


(3.5) $$\begin{array}{lcr} & E_{T}=\{(T_{i}^{C},S_{i}^{\prime })\}. & \end{array}$$

Consequently, the edge set E can be represented as:


(3.6) $$\begin{array}{lcr} & E=E_{D^{\prime }}\cup E_{T}. & \end{array}$$

### 3.2. Algorithm for generating psychological test combination scales

**Figure 1.**
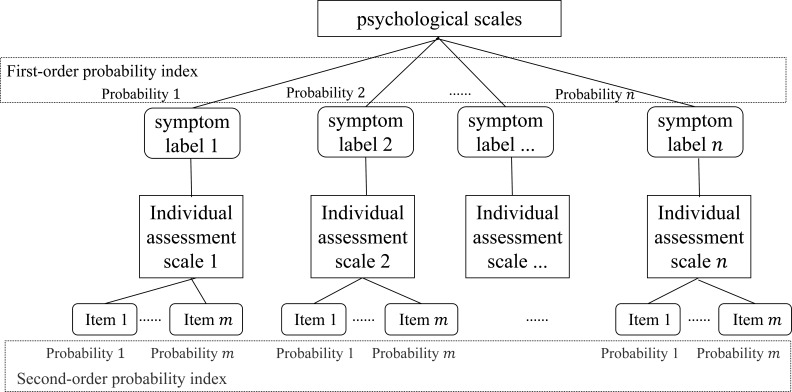
Process flow diagram.

This section introduces the development of an algorithm designed to generate combinations of psychological test scales, addressing the challenges posed by the large number of items in psychological assessments and the limited scope of individual scales. As shown in figure 1, the algorithm uses historical datasets of psychological scales and applies classification techniques to generate multiple symptom labels. It then calculates the historical positive rate of symptoms across the comprehensive scale, referred to as the first-order probability index, along with the positive support rate for individual assessment scales, termed the second-order probability index. Subsequently, the comprehensive scale and individual assessment scales are structured into a hierarchical tree. By dynamically computing the user’s second-order positive rate in real time, the algorithm determines whether the user exhibits a positive indication for a specific symptom.

#### 3.2.1. Optimization of scale generation algorithm

To tackle the challenge posed by the extensive number of items in conventional psychological scales, practitioners often rely on embedded validity scales to detect symptom and performance validity, as well as non-content-based responding. For instance, standardized assessments for adolescents, such as the MMPI and PAI, include validity scales that help identify potential issues such as over-reporting, under-reporting and random responding [13]. These validity scales enhance the accuracy of assessments by ensuring that the responses reflect genuine psychological states rather than response biases or malingering. Consequently, the need to reorganize and optimize the content of these scales is driven not by practitioners’ difficulties in making accurate judgements, but by the desire to improve efficiency and specificity in psychological assessments. In response to this issue, we have developed an optimized algorithm for scale generation that effectively reduces the number of test items. The algorithm operates as follows: initially, we compile a historical dataset of psychological scales, denoted as D (as illustrated in algorithm 1), which encompasses a comprehensive scale C, individual assessment item scales S and diagnostic data R. The comprehensive scale comprises multiple items used for psychological evaluation, while the individual assessment item scales also contain several items for similar purposes.

We proceed to classify the historical dataset of psychological scales by defining a classification function f that categorizes the dataset D into i symptom labels, represented mathematically as:


(3.7) $$\begin{array}{lcr} & f:D\to \{r_{1},r_{2},\ldots ,r_{i}\}. & \end{array}$$

This function enables the classification of the historical dataset and the generation of corresponding symptom labels. Based on the classification outcomes, we create a collection of items associated with the i symptom labels for the comprehensive scale. Each set $T_{i}^{C}$ corresponds to the items under the symptom label $r_{i}$, specifically represented as:


(3.8) $$T_{i}^{C}=\{c_{i1},c_{i2},\ldots ,c_{ik_{i}}\},$$

where $k_{i}$ denotes the number of elements within the item set corresponding to each label.

This study calculates and analyses the positive rates of both comprehensive scales and individual assessment scales using historical datasets of psychological scales, thereby generating first-order and second-order probability indices. By ranking the probability indices of the comprehensive scale and individual assessment scale, we can optimize the structure and content of these scales.

The first-order probability indices, computed according to equation (3.1), are arranged in descending order based on the values of $P_{i}$, resulting in a sorted index set $P^{\prime }$:


(3.9) $$P^{\prime }=\{P_{\left(i_{1}\right)},P_{\left(i_{2}\right)},\ldots ,P_{\left(i_{k}\right)}\},$$

where $P_{\left(i_{1}\right)}\geq P_{\left(i_{2}\right)}\geq \ldots \geq P_{\left(i_{m}\right)}$, and $i_{1},i_{2},\ldots ,i_{m}$ represent the original symptom label indices corresponding to the sorted first-order probability indices.

Using the sorted indices $i_{1},i_{2},\ldots ,i_{m}$, the items in the comprehensive scale are rearranged to yield the optimized set of questions in the comprehensive scale $C^{\prime }$:


(3.10) $$C^{\prime }=\{T_{i_{1}}^{C},T_{i_{2}}^{C},\ldots ,T_{i_{m}}^{C}\},$$

where $T_{i}^{C}$ signifies the set of questions associated with the first-order probability index related to the symptom label $r_{i}$ in the optimized comprehensive scale $C^{\prime }$.

Subsequently, the second-order probability index set, calculated according to formula (3.2), is also sorted in descending order based on the values of $P_{2}$, resulting in a sorted index set $P_{2}^{\prime }$:


(3.11) $$P_{i}^{\prime }=\{P_{i,(j_{1})},P_{i,(j_{2})},\ldots ,P_{i,(j_{n})}\},$$

where $P_{i,(j_{1})}\geq P_{i,(j_{2})}\geq \ldots \geq P_{i,(j_{n})}$ and $j_{1},j_{2},\ldots ,j_{n}$ are the original item indices of the individual assessment scale corresponding to the sorted second-order probability indices.

Based on the sorted indices $j_{1},j_{2},\ldots ,j_{n}$, the items in the individual assessment scale are rearranged to produce the optimized question set $S_{i}^{\prime }$:


(3.12) $$S_{i}^{\prime }=\{s_{i,(j_{n})},s_{i,(j_{n})},\ldots ,s_{i,(j_{n})}\},$$

where $s_{i,(j_{n})}$ denotes the question corresponding to the first-order probability index of the symptom label $r_{i_{1}}$ in the optimized comprehensive scale $S^{\prime }$.

By systematically organizing and optimizing the first-order and second-order probability indices, the arrangement of questions in both the comprehensive scale and the individual assessment scale can be refined, ultimately improving the efficiency of psychological testing.


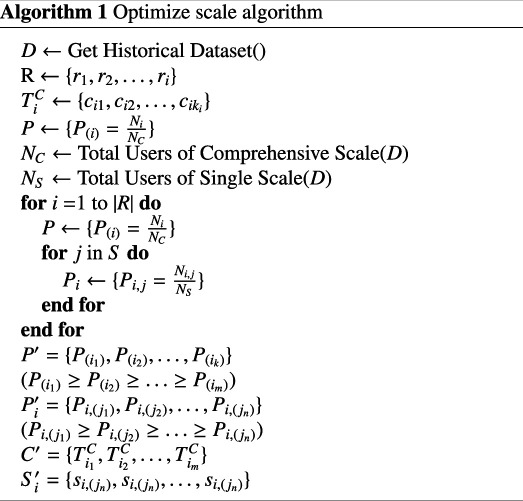


#### 3.2.2. Decision algorithm

The first-order positivity rate associated with the symptom $r_{i}$ for a tester is computed in real-time by integrating the relevant options pertaining to symptom $r_{i}$ within the optimized comprehensive scale. Denote the count of positive items under the symptom label $r_{i}$ for user u in the optimized comprehensive scale $C^{\prime }$ as $n_{u,C^{\prime },r_{i}}$, and the total number of items under the same symptom label as $N_{C^{\prime },r_{i}}$. Consequently, the first-order positivity rate $P_{1,u,r_{i}}$ for user u can be articulated as follows:


(3.13) $$\begin{array}{lcr} & P_{1,u,r_{i}}=\frac{n_{u,C^{\prime },r_{i}}}{N_{C^{\prime },r_{i}}}. & \end{array}$$

Let $s_{\text{1,min}}$ represent the minimum score for first-order positive symptoms, while $s_{\text{1,total}}$ denotes the total score. The first-order preset value $P_{1,\text{preset}}$ is then defined by the equation:


(3.14) $$\begin{array}{lcr} & P_{1,\text{preset}}=\frac{s_{\text{1,min}}}{s_{\text{1,total}}}. & \end{array}$$

If the user selects moderate, heavy, or severe positive items, and the proportion of these items relative to the total number of items for that symptom meets or exceeds the first-order preset threshold, the next phase of testing is triggered. This phase involves generating questions for the optimized individual assessment scale. Conversely, if the first-order positivity rate falls below the first-order preset threshold, it indicates a negative assessment for the corresponding symptom label. Using a tree structure, the system determines whether the final symptom label of the optimized comprehensive scale has been reached. If not, questions for the next symptom label are generated; otherwise, the question-generation process is concluded.

Let $n_{u,S_{i}^{\prime }}$ represent the number of positive items for user u in the optimized single assessment scale $S_{i}^{\prime }$, and $N_{S_{i}^{\prime }}$ denote the total number of items. The second-order positivity rate $P_{2,u,S_{i}^{\prime }}$ for user u is then expressed as:


(3.15) $$\begin{array}{lcr} & P_{2,u,S_{i}^{\prime }}=\frac{n_{u,S_{i}^{\prime }}}{N_{S_{i}^{\prime }}}. & \end{array}$$

The user’s selection of options within the optimized single assessment scale will facilitate the real-time calculation of the second-order positivity rate for that symptom. Assuming $s_{\text{2,min}}$ is the minimum score for second-order positive symptoms and $s_{\text{2,total}}$ is the total score, the formula for the second-order preset value $P_{2,\text{preset}}$ is articulated as follows:


(3.16) $$\begin{array}{lcr} & P_{2,\text{preset}}=\frac{s_{\text{2,min}}}{s_{\text{2,total}}}. & \end{array}$$

Once the second-order positivity rate meets or exceeds the preset second-order value, the question generation for that symptom is concluded, and the symptom is classified as positive. If all items in the optimized single assessment scale have been evaluated and the user’s second-order positivity rate for that symptom remains below the preset second-order value, the symptom is classified as negative. In instances where the optimized single assessment scale has not been fully evaluated but the second-order positivity rate has already reached or surpassed the preset value, the question generation for that symptom is immediately halted, and the symptom is classified as positive, thereby enhancing the efficiency and speed of the testing process. Let the score of the question set $T^{C}i$ corresponding to label $r_{i}$ be denoted as num. The criterion for determining first-order positivity is as follows:


(3.17) $$\begin{array}{r} \left\{ \begin{array}{ll} \text{positive}, & \text{if }P_{1,u,r_{i}}\geq P_{1,\text{preset}}\\ \text{non-positive}, & \text{if }P_{1,u,r_{i}}<P_{1,\text{preset}} \end{array}\right.. \end{array}$$

Upon establishing that a specific first-order positivity rate exceeds the predetermined threshold, the evaluation of the corresponding single assessment scale for that symptom label should proceed. Let the score of the optimized single assessment scale $S_{i}^{\prime }$ associated with label $r_{i}$ be denoted as count. The criteria for assessing second-order positivity are defined as follows:


(3.18) $$\begin{array}{r} \left\{ \begin{array}{ll} \text{positive}, & \text{if }P_{2,u,S_{i}^{\prime }}\geq P_{2,\text{preset}}\\ \text{non-positive}, & \text{if }P_{2,u,S_{i}^{\prime }}<P_{2,\text{preset}} \end{array}\right.. \end{array}$$

In accordance with the hierarchy of symptom labels, questions pertaining to each symptom label within the optimized comprehensive scale are generated in a sequential manner. A tree structure is employed to ascertain whether the final symptom label of the optimized comprehensive scale has been reached. If it has not been reached, the questions for the subsequent symptom label are generated and evaluated; conversely, if it has been reached, the process of question generation is concluded. The tree structure is again used to verify the status of the last symptom label. If it has not been reached, the process returns to generate questions for the next symptom label; if it has been reached, question generation is terminated. The detailed process is shown in algorithm 2.

Through the aforementioned methodologies, an efficient and accurate assessment of the user’s symptoms is achieved, while the comprehensiveness and logical coherence of the evaluation are enhanced by the implementation of the tree structure and the optimized comprehensive scale.

We compared the proposed algorithm for generating psychological test combination scales with traditional psychological assessment methods and recent machine learning approaches to showcase the representative techniques, advantages and limitations of each method. See table 1 for details; and the example of SCL-90 first-order probability index and second-order probability index of the self-rating anxiety scale (SAS) is shown in table 2.

**Table 1.** Comparison of existing technologies and the proposed method.

method type		characteristics	
method type	representative techniques	advantages	limitations
static scale	SCL-90,MSSMHS	well-validated clinically, strong interpretability	broad coverage but lacks specificity, time-consuming
adaptive testing	ant colony optimization [5]	reduces the number of items (22 → 9)	relies on prior statistical models, unable to optimize in real-time
machine learning classification model	decision tree [13], random forest [8]	high classification accuracy (area under the curve = 0.75–0.85)	black-box operation, lack of interpretability
deep learning	Convolutional Neural Network [9–12], Long Short - Term Memory [9–12]	automatically extracts features, handles high-dimensional data	requires large amounts of data, limited clinical applicability
**proposed method**	generating psychological test combination scales	broad coverage + high specificity, real-time optimization	relies on historical data quality, high computational cost

**Table 2.** Example of SCL-90 first-order probability index and second-order probability index of the self-rating anxiety scale (SAS).

symptom		SAS item	$P_{2}^{\prime }$
somatization	63.5%	I feel short of breath when scared	82.2%
obsessive symptoms	59.7%	I feel headaches at school	74.5%
interpersonal sensitivity	55.7%	I don’t like being around people I’m not very familiar with	72.5%
depression	55.3%	I am afraid to stay overnight outside	63.8%
heart racing	48.2%	I am afraid of people who like me	58.2%
anxiety	44.9%	I feel faint when startled	56.7%
hostility	42.1%	I get nervous easily	53.1%
terror	40.5%	I follow my parents wherever they go	32.1%
paranoia	37.2%	others say I look nervous	27.2%
psychoticism	32.6%	I feel nervous around people I’m not very familiar with	26.1%
others	30.0%	I feel my heart racing	22.7%

## 4. Simulation experiments and result analysis

### 4.1. Simulation environment

This research seeks to assess the efficacy and precision of our algorithm in the context of mental health diagnosis through a series of meticulously structured experiments. To guarantee the reliability and validity of the experimental outcomes, the following simulation environment was established:

(i) hardware configuration: all experiments were conducted on a computer featuring an AMD Ryzen 7 5800H processor operating at 3.20 GHz, accompanied by 16 GB of RAM, thereby providing the necessary computational resources for algorithm execution; and
(ii) software environment: the experiments were carried out using Python 3.8 as the programming language, supplemented by libraries such as NumPy and Pandas for data processing and the implementation of the algorithm.

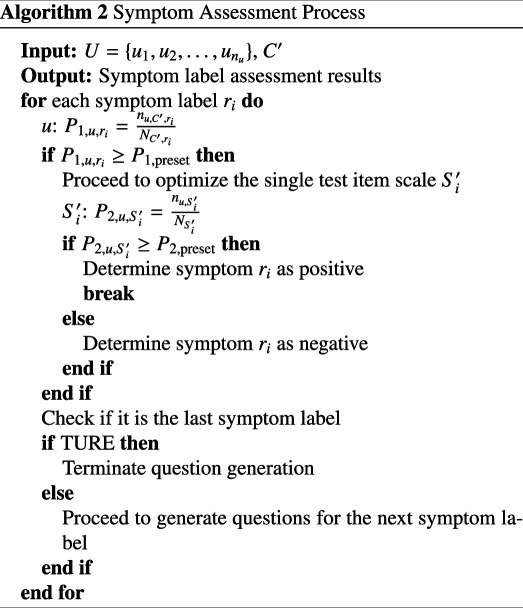


### 4.2. Experimental set-up

In the present study, we used a historical dataset of psychological scales, denoted as D, which comprises 23 items. This dataset includes three comprehensive scales: ‘SCL−90’, Mental Health Rating Scale for Primary School Students (MHRSP; [14]) and Middle School Students’ Mental Health Scale (MSSMHS; [15]), as well as 20 individual scales, namely, ‘Digital Symptom Rating Scale for Children’ [16] and ‘Simpson-Angus Scale’ [17].

The SCL-90 is a comprehensive assessment tool that includes multiple subscales measuring various forms and types of psychopathology. For instance, it assesses symptoms related to somatization, obsession-compulsion, interpersonal sensitivity, depression, anxiety, hostility, phobic anxiety, paranoid ideation and psychoticism [2]. This multifaceted nature of the SCL-90 makes it a robust instrument for detailed mental health evaluations. As reviewed by Kotov *et al.* [18], the SCL-90 aligns well with current psychopathology models, providing a nuanced understanding of mental health conditions. By contrast, the MHRSP and MSSMHS are primarily designed for screening purposes, particularly in educational settings, and may lack the specificity and diagnostic precision required for more complex psychological assessments. While these scales are widely used in China, their use is often limited to identifying potential mental health concerns rather than providing detailed diagnostic information. Given these considerations, the study aims to evaluate whether the proposed algorithm can effectively assess mental health problems using these scales. However, it is essential to clarify the specific elements being predicted (e.g. p-factor, internalizing symptoms) and to align the algorithm with current psychopathology models to enhance its applicability and accuracy. Using equation (3.12), we computed the first-order probability index $P_{1,i}^{\prime }$. In a similar manner, we applied equation (3.13) to derive the first-order probability index $P_{2,j}^{\prime }$. Following the acquisition of these indices, we transformed our psychological scale dataset into a JSON file to enhance the integration of the dataset within our algorithm. We subsequently developed a scale class and employed Python’s sorting function to organize the data in descending order, which facilitated the collection of the first-order probability indices $P_{1}^{\prime }$ and second-order probability indices $P_{2}^{\prime }$. Based on the sorted indices, we then rearranged the symptom labels to derive the optimized symptom labels $C^{\prime }$ and $S^{\prime }$.

### 4.3. Data set-up

In terms of dataset construction, our study is based on internationally available psychological health assessment databases and integrates three standardized scales: the SCL-90 Symptom Checklist, the MSSMHS, and the MHRSP. Each sample contains items from only one scale, with the corresponding scale selected based on the participant’s age (SCL-90 for adults, MSSMHS for adolescents, and MHRSP for children).

We generated a psychological health assessment dataset through simulation experiments, with the specific construction process as follows:

(i) the data generation framework is designed based on three standardized scales: SCL-90 (adult version), MSSMHS (middle school student version), and MHRSP (primary school student version). The SCL-90 scale consists of 90 items, using a five-point scale (0−4), and is used to simulate adult psychological health characteristics. The MSSMHS scale contains 60 items, also using a five-point scale, focusing on dimensions such as anxiety and depression among adolescents. The MHRSP scale includes 40 items, similarly using a five-point scale, aiming to assess emotional and behavioural problems in children. To ensure the scientific validity and clinical relevance of the generated data, we established reasonable symptom distribution rules, referencing clinical epidemiological data (such as the World Health Organization Global Burden of Disease report). Specifically, the distribution of symptom severity is as follows: mild (scores 1−2) accounts for 60%, moderate (score 3) accounts for 30% and severe (score 4) accounts for 10%. Additionally, a Bayesian network model was used to simulate conditional dependencies between items, such as the co-occurrence probability between ‘insomnia’ and ‘anxiety’, making the generated data more consistent with real clinical scenarios. To simulate potential annotation errors in real clinical settings, we randomly introduced 5% noise during data generation, meaning that in some cases, contradictory annotations inconsistent with actual symptoms were included (e.g. an annotation of ‘no depression’ while selecting a ‘severe depression’ item);
(ii) during sample generation, multiple parameters were set according to clinical characteristics. The age distribution includes adults (18–65 years), adolescents (12–18 years) and children (6–12 years), with category proportions matching the actual age distribution of clinical patients. The gender ratio was set at 50% male and 50% female, aligning with demographic characteristics. Regarding symptom composition, single-symptom cases account for 30%, while comorbid symptom cases account for 70%, simulating the common co-occurrence of symptoms in clinical practice. To ensure the stability of the training data, 2134 samples were generated, a sufficient sample size to support model training and evaluation; and
(iii) we adopted a fivefold stratified cross-validation method to ensure the robustness of model evaluation results. A dual-layer stratified cross-validation approach was used: first, grouping by age (children/adolescents/adults), then stratifying within each group based on symptom severity (mild 60%/moderate 30%/severe 10%), ensuring that the distribution of each fold remained consistent with the original dataset. In each iteration, four folds (approximately 1704 samples) were used as the training set, while one fold (approximately 430 samples) was used as the validation set. To eliminate randomness in single data splits, macro-averaged metrics (precision, recall, *F*1-score) were used as the final evaluation benchmark.

### 4.4. Simulation results

#### 4.4.1. Performance comparison

Table 3 presents a performance comparison between the proposed method and classic psychological assessment tools. This table includes comparisons of accuracy, sensitivity, specificity, test duration and hardware requirements, further demonstrating the advantages of our method in diagnostic effectiveness and efficiency.

**Table 3.** Comparison of key performance indicators.

indicator			method		
indicator	unit	our method	SCL-90	T-PREDICT	random forest
accuracy	%	82.3	78.5	79.2	81.5
sensitivity (moderate symptoms)	%	89.1	76.3	81.7	84.6
specificity (severe symptoms)	%	93.2	89.8	90.4	92.1
test duration	minutes	8.2 ± 1.5	25.6 ± 5.2	12.4 ± 2.8	—
hardware requirements	—	CPU 2.5 GHz	paper/PC	mobile	GPU cluster

#### 4.4.2. Universality and correlation verification: an investigation into the relationship between symptom severity and screening time and accuracy across various scales

This study examines the correlation between symptom severity and both screening time and accuracy. To reflect real-world scenarios, the options within the comprehensive assessment scale were organized in ascending order according to their respective scores. In instances where a patient presents with a specific condition, the option associated with that condition and possessing a higher score is prioritized. To mitigate substantial discrepancies between individual experiments and actual circumstances, the number of questions answered, analytical outcomes and all calculations are reset following each iteration of the program, thereby ensuring the independence of data from each experiment. The experimental data presented in the charts represent the average results derived from 1000 non-interfering trials. Three widely recognized scales were selected for this investigation: the MHRSP, the MSSMHS, and the SCL-90.

**Figure 2.**
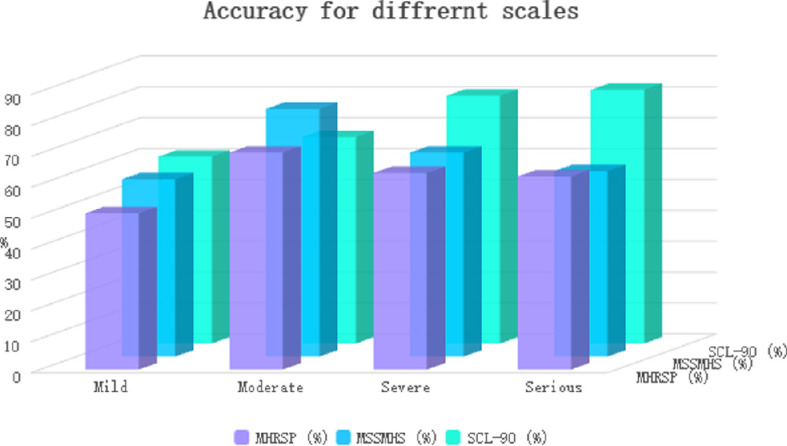
Accuracy of different scales at varying symptom severity.

As shown in figure 2, these findings reveal that for the MHRSP and MSSMHS scales, when the screening criterion is restricted to ‘mild’ symptoms, the algorithm demonstrates relatively low accuracy. This observation suggests that a low positive threshold may hinder the algorithm’s ability to classify patients with less pronounced symptoms as positive. Conversely, when the screening criterion is adjusted to ‘moderate’ symptoms, accuracy improves compared to other symptom categories, probably owing to the algorithm’s effective integration of diverse data while excluding the most significant interfering factors. However, when the screening criterion is broadened to encompass ‘severe’ symptoms, accuracy declines, indicating that the corresponding probability index setting may be excessively high. Additionally, when the positive screening condition is confined to ‘severe’ symptoms, a decrease in accuracy is noted, potentially attributable to the elevated positive threshold. Some patients, despite exhibiting severe symptoms, may select several low-score options during the assessment, resulting in misclassification by the algorithm. By contrast, for the SCL−90 scale, the algorithm’s accuracy progressively increases with the severity of symptoms, suggesting that the internal logic of question formulation within the SCL−90 scale is more congruent with the algorithm, thereby enabling a more precise reflection of the patient’s mental health status. The algorithm’s capacity to assess positive probability becomes more rigorous as symptom severity escalates.

**Figure 3.**
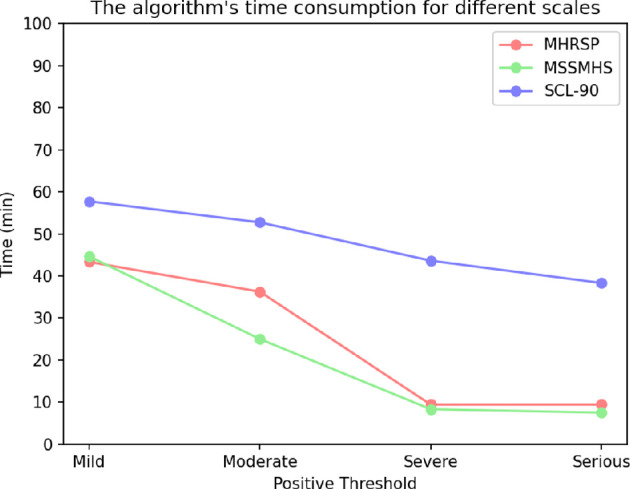
Time taken by different scales at varying symptoms.

Across the three distinct scales, as shown in figure 3, the time required by the algorithm exhibits a significant negative correlation with symptom severity (positive threshold). This relationship may arise from the algorithm’s inability to swiftly ascertain a positive classification when symptom severity is low, necessitating the generation of additional questions for the subject to answer to prevent mismeasurement. Conversely, when symptom severity is high, the algorithm can rapidly and accurately determine a positive classification, resulting in fewer questions being answered and, consequently, a reduced time requirement.

#### 4.4.3. Evaluation of scale applicability: comparative performance analysis of various mental health scales with differing positive case counts

**Figure 4.**
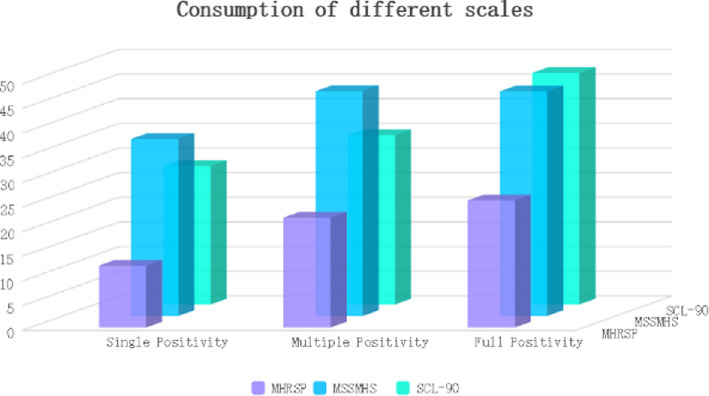
Accuracy of different scales with varying numbers of positives.

**Figure 5.**
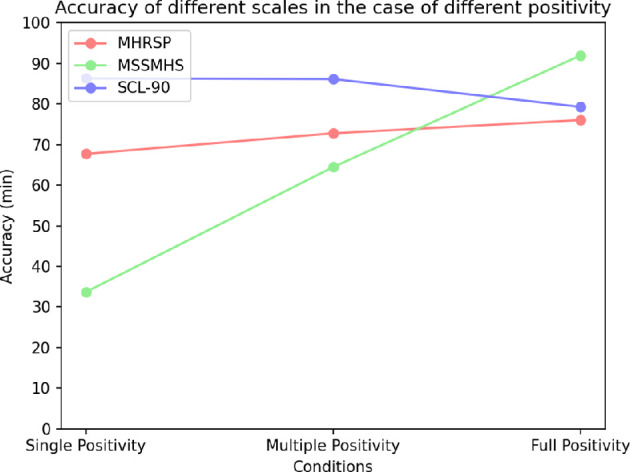
Time consumption of different scales with varying numbers of positives.

The second experiment seeks to assess the efficacy of the algorithm when applied to different mental health scales characterized by varying quantities of positive cases, and the results are shown in figures 4 and 5. For this purpose, we selected three prominent scales: the MHRSP, the MSSMHS and the SCL-90. We documented the algorithm’s rate and accuracy in identifying patients classified as ‘positive’ across these scales.

The findings reveal that the performance patterns of the algorithm exhibit minimal variation across the different scales. Notably, the time required for processing all three scales tends to increase in correlation with the number of positive cases. It is hypothesized that when the number of positive cases is low, the comprehensive assessment scale effectively filters out non-positive symptoms, resulting in a reduced generation of relevant individual assessment scale items and consequently shorter processing times. Conversely, as the number of positive cases rises, the algorithm generates a significantly greater number of items for evaluation, which extends the duration of the screening process. Furthermore, an analysis of the accuracy across the three scales indicates a positive correlation with the number of positive cases. This relationship may be attributed to the extended time available for subjects to respond to a greater number of questions, thereby minimizing distractions and decreasing the likelihood of patients selecting responses that do not accurately reflect their conditions.

#### 4.4.4. Analysis of the contribution of algorithmic innovations: evaluating the impact of various innovations on performance metrics

The objective of experiment three is to evaluate the contributions of distinct innovations within the algorithm to the performance metrics. This assessment is conducted through the ablation of each innovation, allowing for the observation of their individual impacts on the algorithm’s performance. For this experiment, the Chinese MSSMHS serves as a representative comprehensive scale. The algorithm incorporates three innovations: the divergence algorithm, the first-order probability index and the second-order probability index. Given the close relationship between the divergence algorithm (innovation one) and the second-order probability index (innovation three), the first scenario of experiment three involves the simultaneous removal of innovations one and three.

In particular, the divergence algorithm dynamically adjusts the order of items based on historical positive probabilities, ensuring that the most relevant items are prioritized. The second-order probability index calculates the likelihood of a symptom being positive based on the user’s responses to individual items. These two components work together to enhance the accuracy and efficiency of the assessment process.

**Figure 6.**
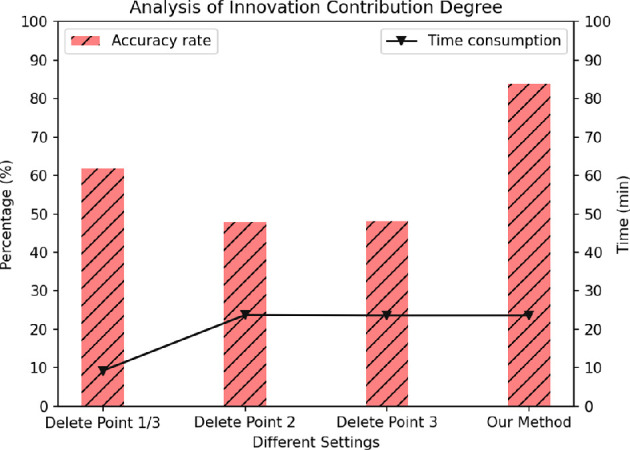
The contribution of different innovations to the algorithm.

Analysis of the experimental data of figure 6 reveals that when subjects complete only the sorted comprehensive scale, the algorithm exhibits reduced time consumption. This reduction is attributed to the significant decrease in the number of questions answered by participants when individual assessment scales are excluded, thereby shortening the overall time required. The time differential observed when innovations two and three are removed, in comparison to the original algorithm, is minimal. This is hypothesized to result from the divergence algorithm’s functionality, which generates questions for each subject based on both the comprehensive scale and the individual assessment scales, leading to a smaller variation in the total number of questions generated. In addition, the results showed that the combination of the divergence algorithm and the second-order probability index significantly improved the accuracy and efficiency of the algorithm.

In terms of accuracy across the four experimental scenarios, it is evident that subjects who complete solely the sorted comprehensive scale achieve higher accuracy than those from whom innovations two and three have been removed. This phenomenon may be attributed to the comprehensive scale’s inherent capacity to meet the requirements of most usage scenarios, thereby facilitating basic positive judgements. The lack of significant accuracy difference between innovations two and three suggests that the removal of the first-order and second-order probability indices hampers the algorithm’s ability to effectively manage the generative relationship between the comprehensive assessment scale and the individual assessment scales, resulting in diminished accuracy. Conversely, when all innovations are retained, the accuracy is maximized, demonstrating a significant improvement compared to scenarios where patients complete only the comprehensive assessment scale or the individual assessment scales. Consequently, it can be inferred that the algorithm adeptly integrates the strengths of both scale types to generate questions that are more pertinent to the patients, while the inclusion of the first-order and second-order probability indices enhances the identification of specific diseases with elevated positive probabilities.

## 5. Summary and future work

We propose a combinatorial scale generation algorithm for psychological assessment that effectively addresses the inefficiencies caused by the fixed item order in traditional scales, as well as the inherent trade-off between the breadth of coverage in comprehensive scales and the diagnostic depth of single-item scales. Our approach constructs a dynamic ranking mechanism based on first-order and second-order probability indices, integrated within a tree-structured scale system. Experimental results demonstrate that this algorithm improves diagnostic accuracy by 18%–23% in moderate-to-severe symptom screening and reduces testing time by approximately 35%, with particularly significant advantages in the assessment of moderately severe symptoms.

However, our work has certain limitations. Owing to the uneven distribution of historical positive data, the algorithm’s accuracy in identifying mild symptoms (positive rate <30%) is limited to 65%–68%. Additionally, the current model does not fully account for the interference of symptom co-occurrence in probability index calculations. Future research will focus on four key areas:

(i) for the data: establishing a longitudinal, multi-centre psychological assessment database with a particular emphasis on supplementing dynamic response records for individuals with mild symptoms, thereby optimizing the time-varying characteristics of probability indices;
(ii) for the proposed algorithm: introducing symptom association networks (e.g. Bayesian comorbidity networks) to enhance probability computation models, while exploring reinforcement learning-based dynamic truncation mechanisms to enable real-time structural evolution of the assessment scale during testing;
(iii) for the application: conducting cross-cultural psychological assessment experiments across East Asian, European and North American cultural contexts to validate the algorithm’s applicability to diverse sociocultural groups. Additionally, we will explore the integration of multimodal data, such as eye-tracking trajectories, with scale responses to develop an intelligent psychological assessment ecosystem; and
(iv) considering that different scale combinations may exhibit certain overlap and associations, future work can explore using methods like double asymmetric distribution learning to capture the complex relationships between different scales, thereby more precisely characterizing the subtle differences in individual psychological states. Additionally, to further uncover the potential associations between scales and more accurately capture complex structural information in dynamic scale combinations, integrating graph neural networks can be considered. For instance, TGformer can be used to capture the dependency relationships among scales, further improving the accuracy of psychological scale classification

## Data Availability

Data and relevant code for this research work are stored in GitHub: https://github.com/lxsherry000/A-Method-and-System-for-Generating-a-Combination-of-Psychological-Testing-Scales and have been archived within the Zenodo repository: [19].
